# Predictors of acute admission of more than 48 hours duration

**DOI:** 10.1186/1757-7241-21-S2-A41

**Published:** 2013-09-09

**Authors:** Erlend Aabel, Christer Aas Hansen, Christian Backer Mogensen

**Affiliations:** 1 Faculty of Health Sciences, University of Southern Denmark, Denmark; 2 Akutforskningsenheden, Sygehus Sønderjylland, Aabenraa, Denmark

## Background

When a patient is admitted to an Emergency Department (ED) in the Region of Southern Denmark, estimation is required whether the patient is expected to remain hospitalized for more or less than 48 hours. If the length of stay (LOS) is expected to be less than 48 hours, the patient stays in the ED until discharge. If LOS is expected to exceed 48 hours, the patient is transferred to the relevant department. The aim of this study was to investigate if information available at admission could be used as predictors of LOS, and to determine which group of staff (nurses, junior physicians or senior physicians) was best able to estimate the correct LOS.

## Methods

A prospective cohort study with collection of data on admitted patients in the ED Aabenraa over a period of 35 days. Information on admission was collected including age, comorbidities (Charlson score), sociodemographic factors, alcohol consumption and smoking habits. The ED staff was asked to give their prediction of LOS. The main outcome was LOS. Data was collected on a total of 730 patients. Analysis was performed using Chi-square test, Kruskal-Wallis test and multivariable logistic regression.

## Results

Significant predictors of LOS exceeding 48 hours were age >80 years (OR 2.69; CI 1.28-5.67), Charlson score 1-2 (OR 2.04; 95% CI 1.05-3.97) and Charlson score >=3 (OR 5.55; 95% CI 2.24-13.78). Senior physicians had the highest accuracy (77%) for LOS and an OR of 8.18 (95% CI 1.92-34.78). Sensitivity was low for all staff, with senior physicians having the highest (56.7%). There was no statistically significant difference in correct estimation of LOS between nurses and junior physicians.

## Conclusion

A general underestimation of LOS was observed among all staff. The estimation of expected length of stay should be assigned to senior physicians. Age and Charlson score can be included in a clinical prediction model to aid the estimation.

